# Density-tunable conjugation of cyclic RGD ligands with polyion complex vesicles for the neovascular imaging of orthotopic glioblastomas

**DOI:** 10.1088/1468-6996/16/3/035004

**Published:** 2015-05-20

**Authors:** Wataru Kawamura, Yutaka Miura, Daisuke Kokuryo, Kazuko Toh, Naoki Yamada, Takahiro Nomoto, Yu Matsumoto, Daiki Sueyoshi, Xueying Liu, Ichio Aoki, Mitsunobu R Kano, Nobuhiro Nishiyama, Tsuneo Saga, Akihiro Kishimura, Kazunori Kataoka

**Affiliations:** 1 Department of Bioengineering, Graduate School of Engineering, The University of Tokyo, 7-3-1 Hongo, Bunkyo-ku, Tokyo 113-8656, Japan; 2 Center for Disease Biology and Integrative Medicine, Graduate School of Medicine, The University of Tokyo, 7-3-1 Hongo, Bunkyo-ku, Tokyo 113-0033, Japan; 3 Molecular Imaging Center, National Institute of Radiological Sciences, 4-9-1 Anagawa, Inage-ku, Chiba 263-8555, Japan; 4 Department of Pharmaceutical Biomedicine, Graduate School of Medicine, Dentistry, and Pharmaceutical Science, Okayama University, 1-1-1 Tsushima-naka, Kita-ku, Okayama 700-8530, Japan; 5 Polymer Chemistry Division, Chemical Resources Laboratory, Tokyo Institute of Technology, R1-11, 4259 Nagatsuta, Midori-ku, Yokohama 226-8503, Japan; 6 Department of Applied Chemistry, Faculty of Engineering, Kyusyu University, 744 Moto-oka, Nishi-ku, Fukuoka 819-0395, Japan; 7 Department of Materials Engineering, Graduate School of Engineering, The University of Tokyo, 7-3-1 Hongo, Bunkyo-ku, Tokyo 113-8656, Japan; 8 Innovation Center of Nanomedicine, Kawasaki Institute of Industry Promotion, 66-20 Horikawa-cho, Saiwai-ku, Kawasaki 212-0013, Japan

**Keywords:** drug delivery, polymersome, cyclic RGD, glioblastoma, MRI (magnetic resonance imaging)

## Abstract

Introduction of ligands into 100 nm scaled hollow capsules has great potential for diagnostic and therapeutic applications in drug delivery systems. Polyethylene glycol-conjugated (PEGylated) polyion complex vesicles (PICsomes) are promising hollow nano-capsules that can survive for long periods in the blood circulation and can be used to deliver water-soluble macromolecules to target tissues. In this study, cyclic RGD (cRGD) peptide, which is specifically recognized by *α*_V_*β*_3_ and *α*_v_*β*_5_ integrins that are expressed at high levels in the neovascular system, was conjugated onto the distal end of PEG strands on PICsomes for active neovascular targeting. Density-tunable cRGD-conjugation was achieved using PICsomes with definite fraction of end-functionalized PEG, to substitute 20, 40, and 100% of PEG distal end of the PICsomes to cRGD moieties. Compared with control-PICsomes without cRGD, cRGD-PICsomes exhibited increased uptake into human umbilical vein endothelial cells. Intravital confocal laser scanning microscopy revealed that the 40%-cRGD-PICsomes accumulated mainly in the tumor neovasculature and remained in the perivascular region even after 24 h. Furthermore, we prepared superparamagnetic iron oxide (SPIO)-loaded cRGD-PICsomes for magnetic resonance imaging (MRI) and successfully visualized the neovasculature in an orthotopic glioblastoma model, which suggests that SPIO-loaded cRGD-PICsomes might be useful as a MRI contrast reagent for imaging of the tumor microenvironment, including neovascular regions that overexpress *α*_V_*β*_3_ integrins.

## 1. Introduction

Self-assembling hollow capsules of polymeric materials, such as polymersomes [1, 2], have been extensively studied as nanocarriers for drug delivery systems (DDS), owing to their loading ability of various therapeutic and diagnostic materials into the internal aqueous phases. Recently, we have developed a novel class of polymersomes, termed ‘polyion complex (PIC) vesicles (PICsomes)’, which are constructed by the self-assembly of polyethylene glycol (PEG)-based block aniomers and homo-catiomers through electrostatic interaction [3–9]. Compared with other polymeric hollow capsules [1, 2], PICsomes can be simply prepared in an aqueous medium just by mixing component polyanions and polycations in a suitable ratio without any additional treatments [3], and their stability was flexibly adjustable by cross-linking modifications [6, 7]. Taking these synthetic advantages, superparamagnetic iron oxide (SPIO)-loaded PICsomes were prepared, and *in vivo* imaging was successfully achieved against hypervascular solid tumors (such as mouse colon cancer: Colon-26) [7] by the enhanced permeability and retention (EPR) effect [5–7, 10, 11]. However, PICsome accumulation through the EPR effect may not work at the location with a tight vascular tumor, such as glioblastoma. A feasible strategy to overcome this issue of PICsome accumulation is the use of ligands that selectively target the neovasculature around tumor sites and the facilitation of the translocation of PICsomes across the endothelium of the tumor vasculature. Nevertheless, strategies and methods for achieving the introduction of ligands to PICsomes are still to be identified. Among ligand molecules, the cyclic RGD (cRGD) peptide might fulfill neovasculature targeting and vascular translocation requirements. It may do so because the cRGD peptide is known to specifically bind to *α*_V_*β*_3_ and *α*_v_*β*_5_ integrins on tumor vasculatures [12–16]. A recent report revealed that cRGD-linked nanocarriers promote active transport (e.g., transcytosis) across tumor vascular barriers [17]. Moreover, although many cRGD-linked nanocarriers have been used in DDS [18–26] and the effect of multivalent binding between cRGDs and their receptors has been investigated by comparing the presence and absence of cRGD moieties [18, 20, 22–26], the specific relationships between cRGD density, their targeting abilities, and materials properties such as carrier size remain controversial.

In this study, we first demonstrate the chemical modification of the PICsome surface to introduce a ligand molecule. Then, we conjugated the cRGD peptide with the distal ends of the PEG strands covering the PICsome surface by a well-controlled chemical procedure. Next, we developed density-tunable cRGD-linked PICsomes, including 20, 40, and 100%-cRGD-linked PICsomes as active targetable nanocarriers. To systematically evaluate and compare these PICsomes, we studied their cellular uptake, conducted fluorescence activated cell sorting, determined their blood clearance and tumor accumulation, and made observations using intravital microscopy. We also identified the cRGD-density value that enables the delivery of PICsomes to glioblastomas with minimal accumulation in other tissue. Finally, we encapsulated SPIO inside cRGD-PICsomes to observe the neovasculature of an orthotopic glioblastoma using magnetic resonance imaging (MRI).

## 2. Materials and methods

### 2.1. Materials

Alpha-methoxy-*ω*-amino PEG (MeO-PEG-NH_2_; *M*_n_ = 2400; *M*_w_/*M*_n_ = 1.11; NOF, Tokyo, Japan) was purified using an ion-exchange CM Sephadex C-50 column (GE Healthcare, Buckinghamshire, UK) before use. Alpha-acetal-*ω*-amino PEG (acetal-PEG-NH_2_) was synthesized as described previously [27]. Beta-benzyl-l-aspartate *N*-carboxy-anhydride (BLA-NCA; NOF), 1-ethyl-3-(3-dimethylaminopropyl) carbodiimide hydrochloride (EDC; Tokyo Chemical Industry, Tokyo, Japan), Cyclo[RGDfk(CX-)] (cRGD peptide, X = 6-aminocaproic acid, *∊*-Acp; Peptide Institute, Osaka, Japan), sulfo-Cy3 mono-reactive dye (Lumiprobe, Orlando, Florida, USA), sulfo-Cy5 mono-reactive dye (Lumiprobe), DyLight488 NHS ester (Thermo Fischer Scientific, Waltham, Massachusetts, USA), methylamine solution (Me–NH_2_; 40%; Wako Pure Chemical Industries, Osaka, Japan), sodium hydroxide (NaOH; Koso Chemical, Tokyo, Japan), hydrochloric acid (HCl; Koso Chemical), acetic acid (CH_3_COOH; Nacalai Tesque, Tokyo, Japan), ferucarbotran (SPIO) solution (Resovist^®^, Fujifilm RI Pharma, Tokyo, Japan), Blocking one (Nacalai Tesque), 4% paraformaldehyde (4% PFA; Wako), Hoechst 33342 (Hoechst; Dojindo Laboratories, Kumamoto, Japan), and other reagents were used without further purification. PBS-Tween solution (0.1% v/v PBS-T) was prepared from Dulbecco’s phosphate-buffered saline (D-PBS; Wako) and Tween 20 solution (10% w/v, Wako).

### 2.2. Cell lines and animals

Normal human umbilical vein endothelial cells (HUVECs; Lonza Japan, Tokyo, Japan) were maintained in EGM^™^-2 BulletKit^™^ medium (Lonza) in a humidified atmosphere of 5% CO_2_ at 37 °C. BALB/c nu/nu mice (female, 18–20 g, 6 weeks old) were purchased from Oriental Yeast (Tokyo, Japan). Anti-mouse CD31 (platelet endothelial cell adhesion molecule; PECAM-1) eFluor^®^ 450Cyclo was purchased from eBioscience (San Diego, CA, USA). For MRI experiments, BALB/c nu/nu mice (male, 22–25 g, 8 weeks old) were purchased from Japan SLC (Hamamatsu, Japan). All animal experiments were performed in accordance with the Guidelines for the Care and Use of Laboratory Animals of the University of Tokyo and the National Institute of Radiological Sciences (NIRS).

### 2.3. Preparation of PICsomes

The block aniomer PEG-*b*-poly(aspartic acid) [MeO-PEG-*b*-P(Asp)] and the homo catiomer *n-*butyl-poly([5–aminopentyl]-*α*,*β*-aspartamide) [Bu-P(Asp-AP)] were synthesized as reported previously [4, 6, 8]. To synthesize end-acetal functionalized PEG-*b*-poly(aspartic acid) [acetal-PEG-*b*-P(Asp)], BLA-NCA was polymerized using an acetal-PEG-NH_2_ macroinitiator to yield end-acetal functionalized PEG-*b*-poly(*β*-benzyl-l-aspartate) (acetal-PEG-*b*-PBLA). Acetal-PEG-*b*-P(Asp) was then produced from acetal-PEG-*b*-PBLA by removing the benzyl group using 0.1 N NaOH.

Block aniomer solution (1.0 mg mL^−1^; a mixture of MeO-PEG-*b*-P(Asp) and acetal-PEG-*b*-P(Asp) at a 75:25 molar ratio) and Bu-P(Asp-AP) (1.0 mg mL^−1^) were prepared separately in 10 mM phosphate buffer (PB, pH 7.4). Both polymer solutions were mixed and vigorously stirred to form PICsomes using a vortex mixer (Scientific Industries, New York, USA). To obtain cross-linked PICsomes, 10 mg mL^−1^ EDC solution (10 eq. versus the -COOH group in the block aniomers) was added to the PICsome solution, and the reaction was continued overnight. The resulting solution was purified by filtration (Vivaspin 6, molecular weight cutoff (MWCO): 300 000; Sartorius Stedim Biotech GmbH, Goettingen, Germany), and the acetal groups were then de-protected at pH 2.0 using 0.1 N HCl to yield aldehyde-functionalized cross-linked PICsomes. The solution was then neutralized by adding 0.1 N NaOH drop-wise, and the pH was maintained at 5.5. Finally, cRGD peptide (10 eq. versus the aldehyde group on the PICsomes) was added, and the reaction mixture was gradually frozen and thawed [28] to facilitate the formation of a thiazolidine ring between the *N*-terminal cysteine and the aldehyde group that converted from the acetal group [26] on the PICsomes’ surface. The unbound cRGD was removed by filtration (Vivaspin 6, MWCO: 300 000), and the cRGD introduction rates were analyzed with ^1^H nuclear magnetic resonance (NMR) spectroscopy (solvent, D_2_O; 80 °C; JEOL LNM-ECS 400, JEOL, Tokyo, Japan) by comparing the relative peak integration values of the aromatic protons in the cRGD residues (7.2–7.5 ppm) with those of the internal standard (0 ppm, 1% wt sodium 3-(trimethylsilyl)-1-propanesulfonate: TMS), which are estimated to be 23.4% (20%-cRGD PICsomes). Other characteristics such as diameter, polydispersity index (PDI), and morphology of obtained PICsomes were confirmed using dynamic light scattering (DLS; Zetasize Nano-ZS instrument, Malvern Instruments, Malvern, UK) and transmission electron microscopy (TEM; JEM-1400 electron microscope, JEOL, Tokyo, Japan). The control PICsomes (Ctrl-PICsomes), and the 40 and 100% cRGD-conjugated PICsomes (40 and 100%-cRGD PICsomes) were prepared as described above, with slight alterations made to the molar ratios of the MeO-PEG-*b*-P(Asp) and acetal-PEG-*b*-P(Asp) from 75/25 to 100/0 for the Ctrl-PICsomes, 50/50 for the 40%-cRGD PICsomes, and 0/100 for the 100%-cRGD PICsomes. The cRGD introduction rates were estimated to be 40.4% for the 40%-cRGD PICsomes, and 97.5% for the 100%-cRGD PICsomes by NMR. The number of cRGD peptides on a single PICsome (*N*_cRGD_) was determined with fluorescence correlation spectroscopy (FCS) using a Zeiss LSM 510 META equipped with the FCS setup ConfoCor 3 (Carl Zeiss, Germany). The values of *N*_cRGD_ were estimated to be ca. 6400 for the 100%-cRGD PICsomes, ca. 2600 for the 40%-cRGD PICsomes, and ca. 1500 for the 20%-cRGD PICsomes (see supporting information: table S1). For fluorescence imaging, the *N*-termini of the block aniomers were labeled with Cy3 (for *in vitro* experiments), Cy5 (for *in vivo* and intravital confocal laser scanning microscopy (IVCLSM) experiments), and DyLight488 (for IVCLSM) as described previously [17, 29, 30], and used for PICsome preparations. More detailed information is provided in the supporting information.

### 2.4. Cellular uptake and *in vitro* CLSM analysis of cRGD-PICsomes

HUVECs (2.5 × 10^3^/well) were seeded into 96-well plates. After 24 h incubation, the media were replaced with fresh medium (100 *μ*L) containing 10 *μ*g (based on the content of the block aniomers) of Cy3-labeled cRGD-PICsomes or Cy3-labeled Ctrl-PICsomes. Subsequently, the cells were incubated for 1, 3, 6, and 24 h, and the media were then replaced with fresh media (100 *μ*L) containing Hoechst (1 *μ*g). After a 5 min incubation, the cells were washed again with fresh media, and the cellular uptake of the PICsomes was evaluated by high-throughput automated analysis of fluorescence images using an IN Cell Analyzer 1000 (GE Healthcare). Differences in the internalization rate of cRGD-conjugated and control PICsomes were assessed using two-way analysis of variance. Data analyses were performed using GraphPad Prism version 5.04 (GraphPad Software, La Jolla, CA, USA). For *in vitro* CLSM, HUVECs were seeded into 35 mm glass-base dishes (2.5 × 10^4^/dish) and treated in the same manner as described for the high-throughput automated analysis. Fluorescence images of live cells were captured using a Zeiss 780 LSM equipped with a 63 × 1.4 numerical aperture plan apochromat oil immersion objective (Carl Zeiss, Oberkochen, Germany). Images of each dish were captured after a 24 h incubation using excitation and emission wavelengths of 535 and 575–625 nm, respectively.

### 2.5. Inhibition of cellular uptake of cRGD-PICsomes *in vitro* using echistatin

HUVECs (1.5 × 10^5^/well) were seeded into 6-well plates. After 24 h incubation, the media were replaced with 2 mL of fresh media containing 0.11 *μ*g of echistatin (Tocris Bioscience, Bristol, UK), a potent irreversible *α*_V_*β*_3_ integrin antagonist. After 1 h, 200 *μ*g (based on the content of the block aniomers) of PICsomes in 10 *μ*L of D-PBS was added, and the cells were incubated for 3 h. Cells were washed three times with D-PBS, and 300 *μ*L of trypsin was added and quickly removed. After 1 min, cells were collected by flushing with media, and centrifuged. The supernatants were then removed, and the cells were resuspended in 1 mL of D-PBS. Control samples were prepared in the same manner, but without the addition of echistatin. Cellular uptake of the PICsomes was then evaluated using flow cytometry (BD LSR II, BD Biosciences, San Jose, CA, USA).

### 2.6. Plasma clearance and accumulation in tumors

To investigate the *in vivo* behavior of cRGD-PICsomes and Ctrl-PICsomes, female BALB/c nude mice aged 8 weeks (*n* = 4) were inoculated subcutaneously with U87MG human glioblastoma cells (1 × 10^8^ cells mL^−1^, 50 *μ*L/mouse) [17]. After 20 days, 1 mg/mouse Cy5-labeled PICsomes (based on the content of the block aniomers) was intravenously (i.v.) injected via the tail vein. The mice were sacrificed after 1, 3, 6, and 24 h, and blood was obtained from the inferior vena cava, heparinized, and centrifuged to obtain the plasma. Tumors and livers were then excised, homogenized, and centrifuged, and the supernatants were collected. The fluorescence intensity of these samples was analyzed using an IVIS imaging system (Xenogen Corporation, Alameda, CA, USA).

### 2.7. Intravital confocal laser scanning microscopy

IVCLSM was performed using a Nikon A1R confocal LSM system attached to an upright ECLIPSE FN1 instrument equipped with a Plan Apo lambda 20 × 0.75 (DIC N2/20×) objective lens (Nikon, Tokyo, Japan), as described previously [17, 29, 30]. All pictures were acquired as spectral images from 10 *μ*m confocal slices. Mixed solutions of DyLight488-labeled 40%-cRGD-PICsomes (5 mg mL^−1^; 100 *μ*L; arbitrary fluorescence units (AFU), 90 000) and Cy5-labeled Ctrl-PICsomes (5 mg mL^−1^; 100 *μ*L; AFU, 12 000) were administered using a tail vein catheter. Images of the tumor vasculature were acquired 1 and 6 h after administration. To confirm the effects of the fluorescent dye, experiments were performed using the opposite combination of fluorescence probes and carriers (Cy5-labeled 40%-cRGD-PICsomes (5 mg mL^−1^; 100 *μ*L; AFU, 12 000) and DyLight488-labeled Ctrl-PICsomes (5 mg mL^−1^; 100 *μ*L; AFU, 90 000)). To accurately analyze the distribution of the cRGD-PICsomes, animals were pre-injected with PECAM-1 (eFluor 450-labeled anti-mouse CD31; 0.2 mg mL^−1^, 50 *μ*L/mouse). Subsequently, Cy5-labeled 40%-cRGD-PICsomes (5 mg mL^−1^; 200 *μ*L/mouse; AFU, 12 000) were administered via a tail vein catheter. Cy5 was excited at 640 nm, and the fluorescence emissions were detected at 662–738 nm. Dylight488 was excited at 488 nm, and the fluorescence emissions were detected at 500–550 nm. PECAM-1 was excited at 405 nm, and the fluorescence emissions were detected at 425–475 nm. Data were processed using Nikon NIS-Elements C software (Nikon, Tokyo, Japan).

### 2.8. Fluorescence immunohistochemistry

To assess the location of the PICsomes, the tumor tissue was excised and fixed in optimal cutting temperature compound (Sakura Finetec Japan, Tokyo, Japan). Next, 10 *μ*m sections were cut and fixed in 4% PFA for 5 min at room temperature (r.t.) and washed with PBS-T for 1 min at r.t. After a single blocking treatment using Blocking one (Nacalai Tesque) for 10 min at r.t., the slides were incubated with monoclonal antibodies against CD31 (1:300 dilution; BD Biosciences) at a temperature of 25 °C for 30 min. They were then washed twice with PBS-T for 5 min at r.t., and incubated with a 1:300 dilution of Alexa Fluor 488-labeled goat anti-rat IgG (H + L) antibodies (Molecular Probes, Eugene, OR, USA) for 15 min at r.t. The nuclei were stained with a 1:300 dilution of Hoechst 33342. Samples were analyzed using CLSM.

### 2.9. Preparation of SPIO-loaded PICsomes

Block aniomer solutions (1.0 mg mL^−1^, mixture of MeO-PEG-*b*-P(Asp) and acetal-PEG-*b*-P(Asp) at 50:50 (mol:mol)) were prepared in 10 mM PB (pH 7.4). The resulting solutions were mixed with a one-tenth volume of diluted SPIO solution (10 mg mL^−1^; 0.52 mg mL^−1^ Fe). A 1.0 mg mL^−1^ Bu-P(Asp-AP) solution was prepared separately in 10 mM PB (pH 7.4). Both polymer solutions were mixed and then stirred vigorously using a vortex mixer to form PICsomes. The cross-linked PICsomes were prepared as described in section 2.3, and SPIO-loaded PICsomes were isolated using preparative gel permeation chromatography (Sephacryl^™^ S-1000, GE Healthcare). For preparation of SPIO-loaded cRGD-PICsomes, the acetal-groups were de-protected and the solutions were neutralized and purified as described in supporting information. The diameter and morphology of the PICsomes were confirmed by DLS and TEM, respectively. The inclusion of SPIO was confirmed by energy dispersive x-ray spectroscopy (EDS) analysis using a JEM-2100F (HC-STEM) system, (JEOL, Tokyo, Japan). In addition, the Fe concentration in the SPIO-loaded PICsomes was determined using inductively coupled plasma-mass spectroscopy (ICP-MS) on an Agilent 7700x ICP-MS instrument (Agilent Technologies, Palo Alto, CA, USA). The N-termini of the block aniomers were labeled with Cy5 for fluorescence imaging [5–7] and were then used for PICsome preparation. More detailed information is provided in the supporting information.

### 2.10. *In vivo* MRI measurements in an orthotopic glioblastoma model

To assess the *in vivo* tumor accumulation of SPIO-loaded cRGD-PICsomes in an orthotopic glioblastoma model, U87MG cells were grafted (5.0 × 10^7^ cells mL^−1^, 2 *μ*L/mouse) by stereotaxic injection into the right caudate and putamen (depth, 3 mm) [17]. Tumors were grown for more than two weeks, and reached volumes >2.0 mm^3^. The mice were initially anesthetized using 3.0% isoflurane (Escain, Mylan, Tokyo, Japan), and were then ventilated using 1.5–2.0% isoflurane and an O_2_:air mixture of 1:1. Polyethylene catheters (PE-10, BD) were placed in the tail vein to administer the drugs, and 2D multi-slice spin-echo (SE) and gradient-echo (GE) images were acquired 1 and 6 h after the i.v. administration of SPIO-loaded cRGD-PICsomes or Ctrl-PICsomes without cRGD ligands (dose = 40 *μ*mol kg^−1^ on a Fe concentration basis).

MR images were acquired using a 7.0 Tesla, 20 cm bore magnet (Bruker-Biospin, Ettlingen, Germany) interfaced with an Avance III system comprising a 2-channel high-sensitivity cooled radio frequency (RF) coil (CryoProbe, Bruker-Biospin). During the *in vivo* MRI experiments, rectal temperatures were monitored using an optical fiber thermometer (FOT-M, FISO Technology, Quebec, Canada), and were maintained at approximately 36.5 ± 0.5 °C using warm air and water circulation. Fast SE imaging for *T*_2_-weighted MRI was acquired rapidly using a relaxation enhancement (RARE) sequence with the following parameters: repetition time (TR)/echo time (TE), 2000/40 ms; field of view, 25.6 × 16.0 mm^2^; matrix = 256 × 160; resolution = 100 × 100 *μ*m; number of slices = 10; slice thickness, 1.0 mm with no slice gap; slice distance, transaxial; RARE factor, 8; and number of acquisition (NEX), 8. Multi-echo SE imaging for transverse relaxation rate (*R*_2_) calculations were then performed using the following parameters: TR/TE, 3000/10–100 ms (10 echoes); NEX, 1; all other parameters were the same as those described above for the fast SE sequence. Multi-echo GE imaging for the *T*_2_∗-weighted MRI was performed as follows: TR/TE, 1000/4–58 ms in steps of 6 ms (10 echoes); flip angle, 30°; NEX, 3; all other parameters were the same as those described above. *R*_2_ maps, which are the reciprocal of the transverse relaxation times (*T*_2_) of water protons, were calculated using image-processing software (MRVision, version 1.6.8, MR vision, Winchester, Massachusetts, USA). The delta *R*_2_ (*ΔR*_2_) maps were calculated as *ΔR*_2_ = the *R*_2_ of the tumor region after the administration of SPIO-loaded PICsomes—the *R*_2_ of the tumor region before administration.

## 3. Results and discussion

### 3.1. Preparation of cRGD-conjugated PICsomes with tunable cRGD densities

**Figure 1.**
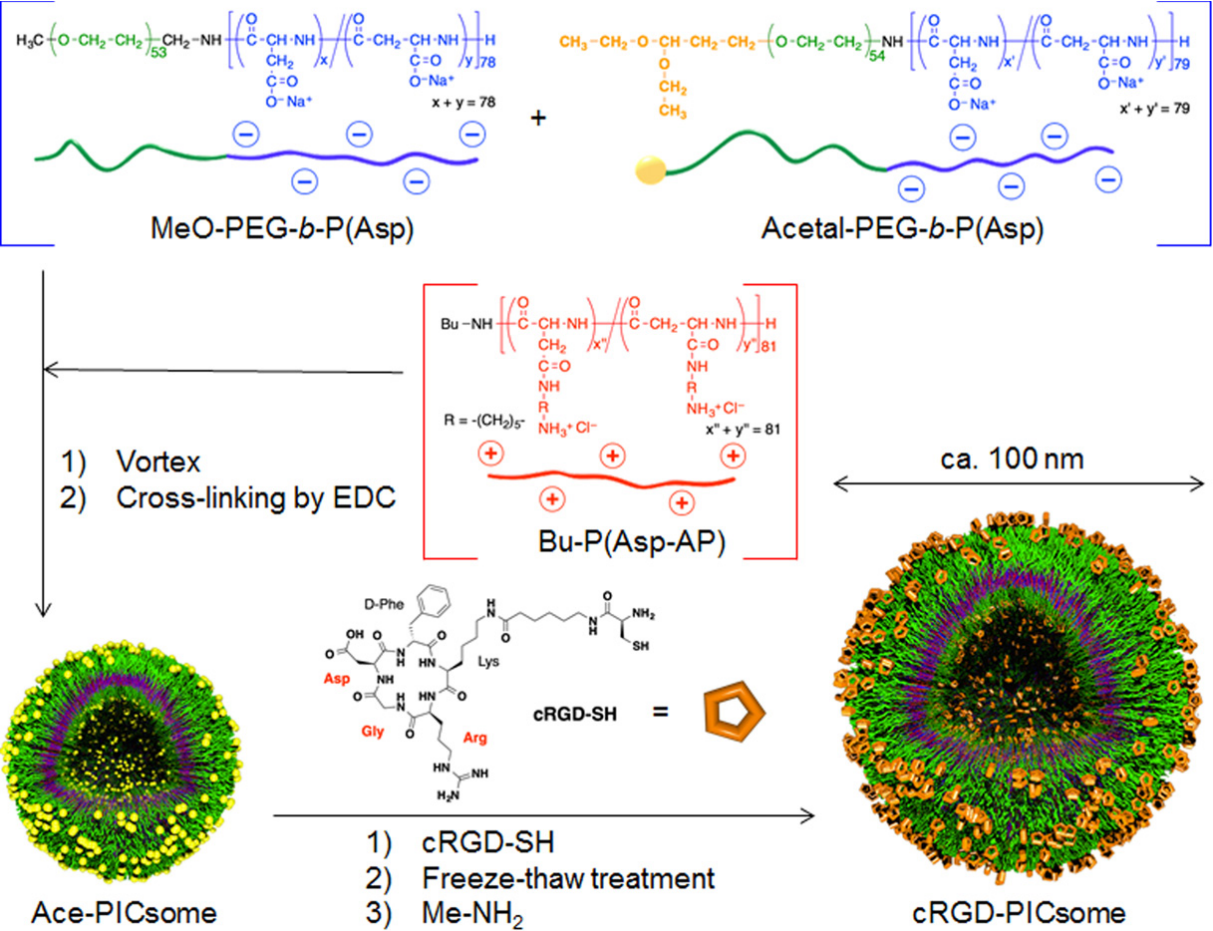
Schematic representation of the protocol used to prepare cRGD-conjugated PICsomes with tunable cRGD densities (yellow spheres, acetal groups; orange pentagons, cyclic RGD ligands).

**Figure 2.**
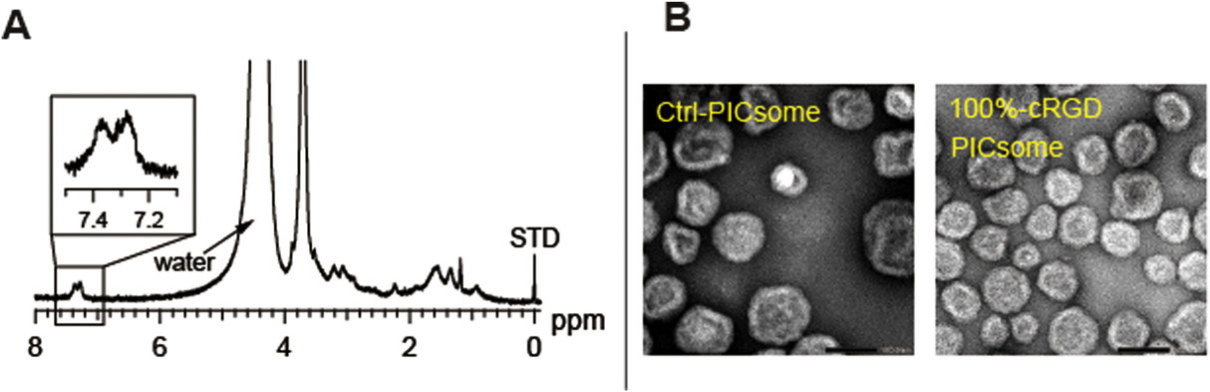
Characterization of the cRGD-PICsomes. (A) ^1^H NMR spectrum of a cRGD-conjugated PICsome (400 MHz, D_2_O, 80 °C), (B) a TEM image of uranyl acetate staining (scale bar, 100 nm).

Cross-linked PICsomes were prepared using an EDC reaction that coupled the carboxylic groups of the block aniomers with the amino groups of homo catiomers in the PICsome membranes. Subsequently, the cRGD peptide was conjugated onto the cross-linked PICsomes via the formation of a thiazolidine ring between the *N*-terminal cysteines of cRGD peptide and the aldehyde groups, which were converted from acetal groups through a deprotection reaction, on the *α*-ends of the PEG strands on the surface of the PICsomes (figure 1). Importantly, ligand molecules need to insert into the outer layer of the PICsomes to facilitate receptor reorganization. However, in some cases, block copolymers that were pre-functionalized with peptides before self-assembly produced undersigned molecular architectures by peptide aggregation [31]. Therefore, the ‘surface modification’ strategy used in the current study should be advantageous for minimizing the above subsidiary subject during PICsome formation. The density of cRGD ligands in the PICsomes was controlled by varying the ratio of MeO-PEG-*b*-P(Asp)/acetal-PEG-*b*-P(Asp) in the precursor acetal functionalized PICsomes (Ace-PICsomes). To facilitate the cRGD conjugation reactions, freezing and thawing was performed in the presence of an excess amount of cRGD (10 eq. per aldehyde group in the block aniomers of the PICsomes; see supporting information) [28]. Excess amount of Me–NH_2_ was added to the PICsome solution to quench any residual aldehyde groups. Finally, the crude solution was purified by filtration to yield cRGD-conjugated and cross-linked PICsomes. Figure 2(A) shows a typical ^1^H NMR spectrum of the resulting cRGD-conjugated PICsomes in D_2_O. The molar percentage of the conjugated cRGD was estimated by comparing the relative peak integration values of the aromatic protons in the cRGD residues (7.2–7.5 ppm) with those in the internal standard (0 ppm, 1% wt TMS) (table 1). Representative characteristics of the cRGD-conjugated PICsomes, including particle size, PDI, zeta potential, and vesicular morphology, were measured using DLS and TEM. The mean PICsome diameter was 86–94 nm, with a relatively narrow dispersity (table 1). TEM analysis revealed that the cRGD-PICsomes formed thin-layer vesicular structures (figure 2(B)), and that the size of the obtained PICsomes was consistent with the DLS results (table 1). In addition, the zeta potentials indicated that cRGD conjugation may not affect the surface charge (table 1). Overall, these data suggest that the preparation of PICsomes with different cRGD densities was achieved without affecting the physical and chemical properties of the PICsomes.

**Table 1.** Characteristics of the obtained cRGD-conjugated PICsomes.

Sample	cRGD content^a^ (mol %)	Diameter^b^ (nm)	Diameter^c^ (nm)	PDI^b^	Zeta potential^d^ (mV)
Ctrl-PICsome	0	92 ± 5	90 ± 12	0.085	−28
20%-cRGD-PICsome	23.4	86 ± 10	82 ± 21	0.094	−27
40%-cRGD-PICsome	40.4	94 ± 8	86 ± 21	0.016	−23
100%-cRGD-PICsome	97.5	92 ± 2	85 ± 23	0.072	−20

a The cRGD content was determined using ^1^H NMR.

b The number average diameter, as determined using DLS (*n* = 10, mean ± s.d., s.d. = standard deviation).

c The number average diameter, as determined using TEM (*n* = 200, mean ± s.d.).

d The zeta-potentials were determined using a zetasizer.

### 3.2. Cellular uptake of cRGD-conjugated PICsomes

**Figure 3.**
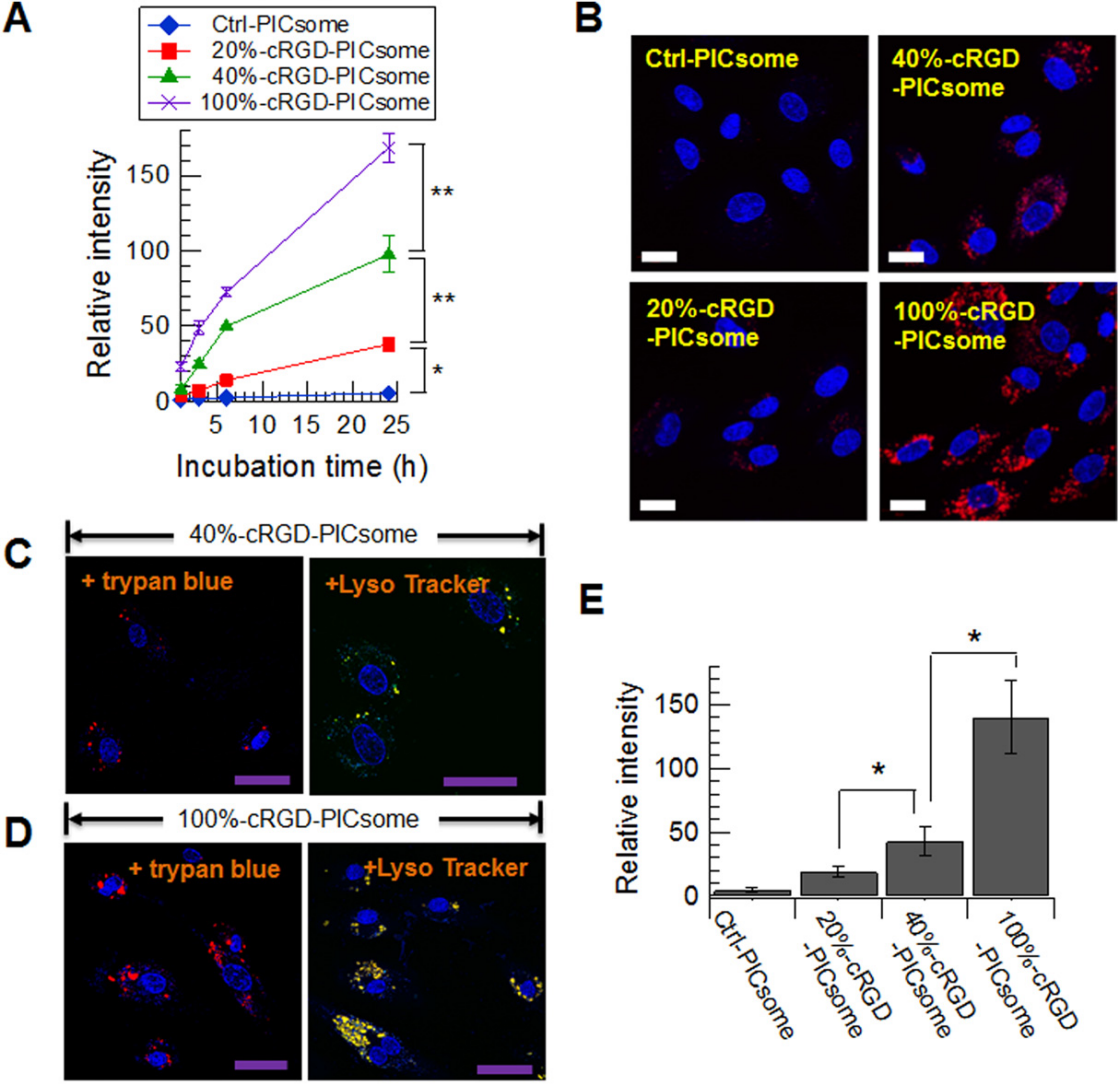
Uptake of cRGD-PICsomes and Ctrl-PICsomes into HUVECs. (A) High-throughput automated analysis of fluorescence images using an IN Cell Analyzer 1000. Data are presented as the mean ± standard error of the mean (s.e.m.), *n* = 3. Two-way ANOVA was used to analyze the differences in the fluorescence intensity, and ∗*P* < 0.005 and ∗∗*P* < 0.001 were considered significant. (B) Images were captured after a 24 h incubation of HUVECs with PICsomes (red) using confocal laser scanning microscopy (CLSM); nuclei were stained using Hoechst 33342 (blue). Scale bar (white) = 20 *μ*m. CLMS images of 40%-cRGD-PICsomes (C) and 100%-cRGD-PICsomes (D); the late-endosomes and lysosomes were stained using Lyso Tracker Green (green). Scale bar (purple) = 50 *μ*m. (E) Quantitative analysis of fluorescence intensity with quenching method using trypan blue. Data are presented as the mean ± standard deviation, *n* = 30. The data were analyzed using Student’s *t*-test, and *P*∗ < 0.001 was considered significant.

The *in vitro* cellular internalization of 20, 40, and 100%-cRGD-PICsomes and Ctrl-PICsomes was evaluated in HUVECs, which overexpress the *α*_V_*β*_3_ and *α*_v_*β*_5_ integrins that recognize cRGD [12–16]. Cellular uptake experiments were performed by the high-throughput automated analysis of fluorescence images using IN Cell Analyzer 1000. Data revealed that the strength of the fluorescence signals increased in proportion to cRGD density (figure 3(A)). In particular, a rapid increase in fluorescence signals was observed in experiments using 40%- (green line in figure 3(A)) and 100%-cRGD-PICsomes (purple line in figure 3(A)) during the initial 6 h incubation, which resulted in a >15-fold higher fluorescence intensity versus Ctrl-PICsomes after 24 h incubation. These results indicate that a high density of cRGD facilitates the binding and/or the internalization of PICsomes. CLSM experiments also revealed the importance of cRGD density. Consistent with the data obtained from the IN Cell Analyzer, the confocal images taken after the 24 h incubation enabled the direct visualization of the density-dependent association of cRGD-PICsomes into the HUVECs (figure 3(B)). Because integrins are transmembrane receptors, it was necessary to verify whether the cRGD-PICsomes were internalized into the cells. Therefore, we performed CLSM experiments with a fluorescence quenching method using trypan blue. As shown in figures 3(C) and (D), the existence of red fluorescence signals (cRGD-PICsomes) were confirmed inside the HUVEC cells. The fluorescence signals at 24 h incubation strengthened with increasing amounts of cRGD (figure 3(E)), which is consistent with the IN Cell Analyzer measurements at 24 h. Moreover, by staining the late-endosomes and lysosomes with Lyso Tracker Green, the co-localizations of cRGD-PICsomes and late-endosomes/lysosomes in HUVECs were observed (figures 3(C) and (D): right panels), indicating that cRGD promoted the cellular uptake of PICsomes.

**Figure 4.**
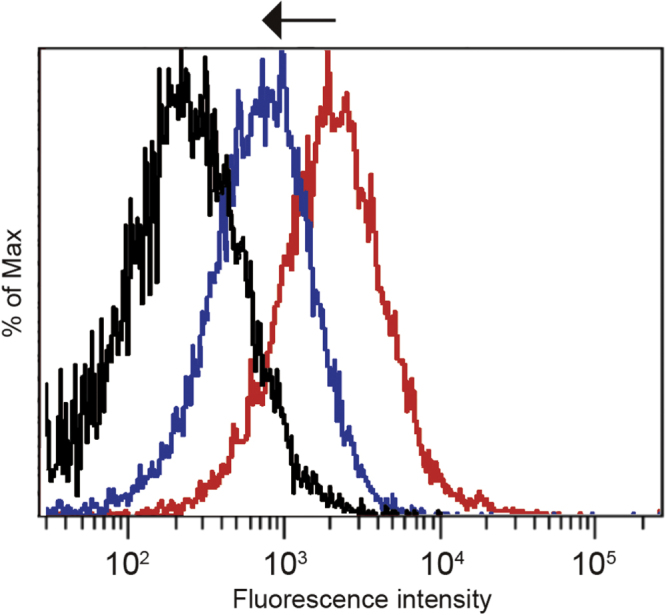
Inhibition of the cellular uptake of cRGD-PICsomes using echistatin in HUVECs. Black line, untreated cells; red line, cells incubated with Cy3-labeled 100%-cRGD-PICsomes; blue line, cells treated with 10 nM echistatin and incubated with Cy3-labeled 100%-cRGD-PICsomes.

Next, we evaluated the binding capacity of cRGD-PICsomes to integrins on the surface of HUVECs by flow cytometry using cellular uptake inhibition assays in the presence of echistatin (figure 4). The 49-amino-acid peptide echistatin belongs to the disintegrin family, and competitively inhibits the binding of cRGD to *α*_V_*β*_3_ integrin, thereby affecting the internalization of cRGD-PICsomes. These competition experiments showed that the fluorescence peak of cells co-incubated with cRGD-PICsomes in the presence of excess echistatin was shifted to the left (figure 4; blue line), indicating reduced binding that most likely resulted from occupation of *α*_V_*β*_3_ integrin by excess free echistatin. These results suggest that integrins play an important role in the cellular uptake of cRGD-PICsomes, and that the 40 and 100% cRGD-PICsomes are candidates for further applications because they have the appropriate density of cRGD ligand on their surface.

### 3.3. Evaluation of cRGD-conjugated PICsomes activity *in vivo*

**Figure 5.**
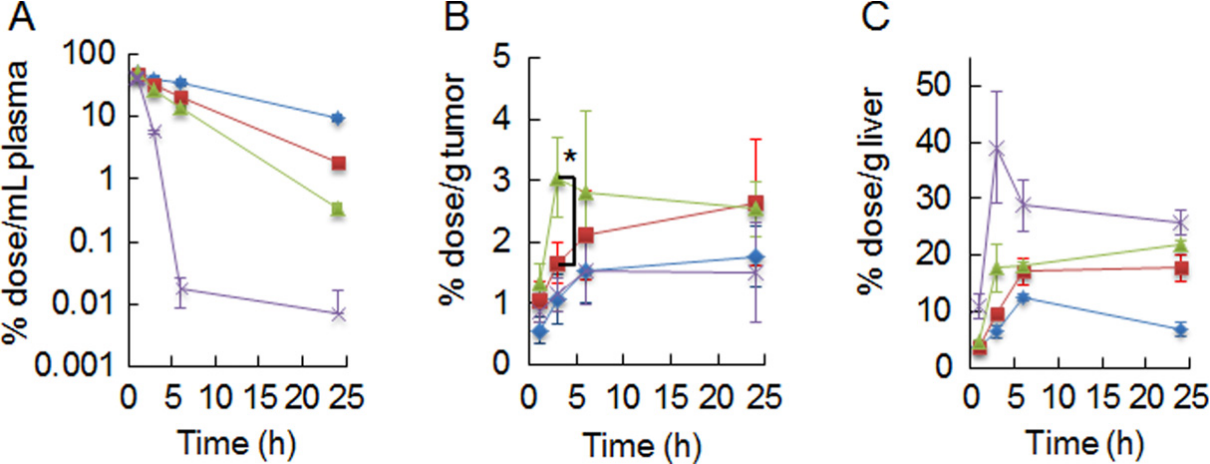
Plasma clearance (A), tumor- (B), and liver- accumulation (C) of PICsomes. Blue line, Ctrl-PICsomes; red line, 20%-cRGD-PICsomes, green line, 40%-cRGD-PICsomes; purple line, 100%-cRGD-PICsomes. The data were analyzed using Student’s *t*-test. Data are presented as the mean ± s.e.m., *n* = 4; ∗*P* < 0.05.

The *in vitro* experiments described in section 3.2 confirmed that the cRGD ligand could recognize *α*_V_*β*_3_ integrin. As such, tumors that have a highly angiogenic vasculature and overexpress integrins, such as glioblastoma, are a suitable model to evaluate the potential of targeting the neovasculature using cRGD-PICsomes. cRGD-PICsomes with varying ligand densities and Ctrl-PICsomes (1 mg/mouse, based on the block aniomer content in the PICsomes) were injected (i.v.) into mice bearing U87MG subcutaneous tumor xenografts, and the mice were sacrificed after 1, 3, 6, and 24 h. As shown in figure 5(A), Ctrl-PICsomes (blue line) were stable in the blood circulation, and approximately 20% of the initial dose was retained in the plasma 24 h after administration. In contrast, the density of the surface cRGD moieties affected the stability of the cRGD-PICsomes in the circulation. Specifically, 100%-cRGD-PICsomes (purple line) were eliminated rapidly (figure 5(A)). It is noteworthy that integrins, including cRGD binding integrins (e.g., *α*_V_*β*_3_ and *α*_5_*β*_1_), are important proteins for promoting cell−cell adhesions and are extant in various organs regardless of their quantities [12–15]. The liver is also equipped with *α*_V_*β*_3_ and/or *α*_5_*β*_1_ integrins [32–35]. Thus, there is a possibility that introducing cRGD might affect the accumulation of PICsomes in the liver. In fact, we observed variations in liver accumulation, particularly when the amount of cRGD was increased (figures 5(C) and (D)). Based on the accumulation profiles, a large quantity of 100%-cRGD PICsomes was captured by the liver within 3 h after injection. This might be a reason for the rapid clearance of the 100%-cRGD PICsomes from the bloodstream. Interestingly, the tumor accumulation of PICsomes was not correlated with their prolonged survival in the bloodstream; more 20%- (red line) and 40%-cRGD PICsomes (green line) accumulated in tumors than the Ctrl-PICsomes (figure 5(B)) did. A comparison of the 20 and 40%-cRGD-PICsomes reveals that the latter exhibited faster accumulation during the initial 6 h after injection (figure 5(B)). Moreover, temporal changes in the tumor-to-blood ratios indicate the utility of the 40%-cRGD-PICsomes until 6 h after their administration (figure S4). Overall, these data suggest that there is a critical balance between ligand density and biodistribution properties in terms of tumor accumulation, and 40%-cRGD PICsomes may thus have the best potential for *in vivo* imaging applications due to their fast and effective accumulation profile. To date, many nanocarriers have been employed in cRGD-mediated targeting to obtain better cancer diagnostic and therapeutic outcomes, because cRGD conjugation imparts the advantage of a specific binding property with nanocarriers. However, our results reveal the nonlinearity between cRGD payload and selectivity and that large liver accumulation should be the core issue in cRGD-mediated DDS. Our findings, relating to the control of cRGD density (i.e., below 40% in PICsomes) might offer the key to being able to circumvent these issues.

### 3.4. Real-time observations of neovascular targeting by cRGD-conjugated PICsomes using IVCLSM

**Figure 6.**
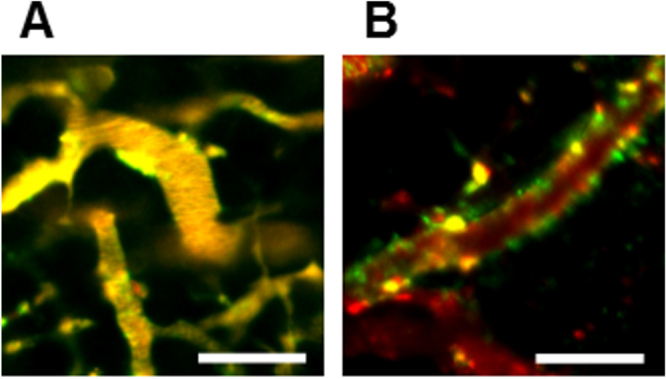
Neovascular-targeting of PICsomes following tail-vein injection. IVCLSM images observed in tumor blood vessels (A) 1 h and (B) 6 h after administration of PICsomes. Red, Cy5-labeled Ctrl-PICsomes; green, DyLight488-labeled 40%-cRGD-PICsomes; yellow, co-localization of red and green signals. Scale bar = 100 *μ*m in all images.

The direct observation of PICsomes *in vivo* can provide information on their biological behavior. Therefore, we used IVCLSM for the real-time analysis of fluorescence-labeled PICsomes in living animals [17, 29, 30]. Ctrl-PICsomes and 40%-cRGD-PICsomes were labeled with Cy5 (red) and DyLight488 (green) fluorescent probes, respectively (see supporting information), and co-injected into U87MG tumor-bearing mice. Figure 6 shows IVCLSM snapshots of the tumor vasculature. One hour after administration, the blood appeared yellow (figure 6(A)), demonstrating that the Ctrl-PICsomes (red) and 40%-cRGD-PICsomes (green) were both circulating stably without aggregation. Interestingly, the 40%-cRGD-PICsomes (green) accumulated in the tumor 6 h after administration and localized around the vessel walls (figure 6(B), green), whereas Ctrl-PICsomes (red) did not selectively accumulate and remained in the circulation (figure 6(B), red). To confirm that these observations were due to specific effects of the cRGD ligand and not artifacts related to the fluorescent dye, the experiment was repeated using the opposite combination of fluorescent probes, which similarly revealed the uptake of cRGD-PICsomes, but not Ctrl-PICsomes (figure S5).

**Figure 7.**
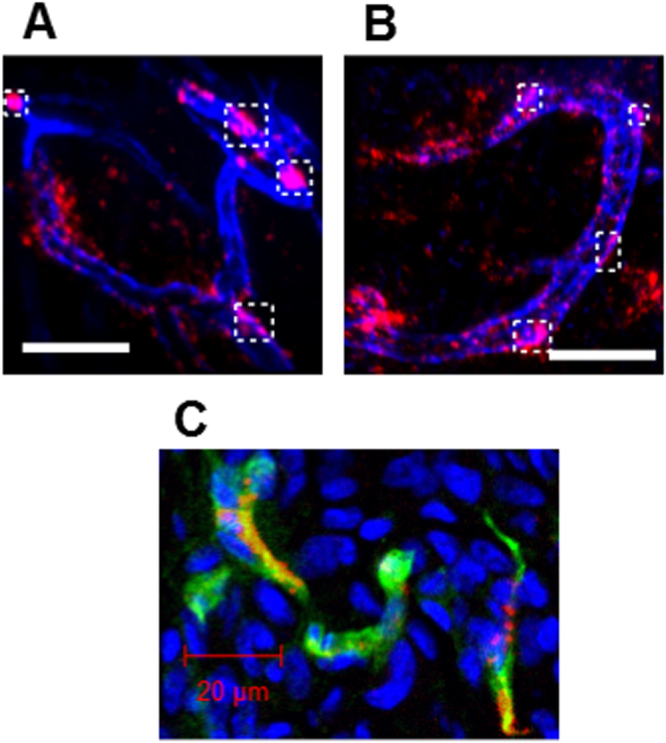
IVCLSM analysis of PICsomes in the tumor vasculature. Blood vessels were pre-stained (blue) with anti-CD31 (PECAM-1) antibodies, and tumor vasculature snapshots were then taken (A) 6 h and (B) 24 h after the administration of 40%-cRGD-PICsomes (red). Scale bar = 100 *μ*m in all images. Co-localizations of blood vessels (blue) and 40%-cRGD-PICsomes (red) in the region selected (indicated by a white rectangle in figures 7(A) and (B)). (C) Confocal laser scanning microscopy (CLMS) analysis of a tumor section. The tumor was harvested 3 h after injection of 40%-cRGD-PICsomes (red). The vasculature was stained with PECAM-1 (green), and nuclei were stained using Hoechst 33342 (blue). Scale bar = 20 *μ*m.

In addition, the neovasculature was pre-stained using anti-CD31 (PECAM-1) antibodies (eFluor 450 Cyclo), which stain the endothelial layers, prior to the administration of Cy5-labeled 40%-cRGD-PICsomes (figure 7). The localization of the 40%-cRGD-PICsomes (red) was then monitored using IVCLSM. The results obtained 6 h after injection clearly revealed co-localized fluorescence signals (magenta) of 40%-cRGD-PICsomes (red) and CD-31 (blue) along the neovasculature (square regions with white dotted lines in figure 7(A)). Even 24 h after injection, the fluorescence signals of 40%-cRGD-PICsomes (red) remained in the neovasculature of the tumors (figure 7(B)). To confirm this observation, immunofluorescence staining of the same tumor was performed. Figure 7(C) confirms the co-localization of 40%-cRGD-PICsomes (red) and PECAM-1 in the tumor vasculature (green), revealing successful neovascular targeting of the cRGD-PICsomes.

Previous studies have also demonstrated IVCLSM of neovasculature by using various cRGD-linked nanocarriers. For example, Schiffelers *et al* showed the rapid adhesion (∼2 min) of cRGD-linked and PEGylated-liposomes to the vessel wall in Lewis lung carcinoma [19]. Hak *et al* observed that cRGD-linked nanoparticles can accumulate to the tumor vasculatures within 10 min after administration [20]. These nanocarriers had similar diameters (ca. 100 nm) as the PICsomes in this study, and reported the importance of cRGD binding to its receptors by comparing the presence and absence of cRGD-ligands, but not cRGD-densities. Furthermore, other research has revealed that large cRGD-linked nanocarriers (ca. 120–320 nm) tend to restrict extravasation through tumor vasculature [18, 21] and small one (ca. 30 nm) penetrate deeper into the tumor parenchyma via transcytosis [17]. In this view, the relationship between ligand density and nanocarrier size should be the crucial factor in restricting the extravasation and/or the cRGD-mediated translocation of nanocarriers across the endothelium of the tumor vasculature. Considering that the diameter of the integrin head is 12 nm [36], it is possible that a single cRGD-linked nanocarrier with less than 12 nm cRGD-cRGD distance and an appropriate size larger than two integrin heads (e.g., more than 24 nm), can simultaneously bind to several integrin molecules when they are overexpressed and closely surrounded (figure S6(A)). To fulfill these conditions, the relationship between ligand density and the size of the nanocarriers must agree to the following equation:

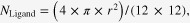
where *N*_Ligand_ is the required number of cRGDs, and *r* is the radius of the nanocarriers. Figure S6(B) shows the calculated relationship. For example, according to this calculation, the 100 nm nanocarriers required over 218 cRGD molecules on their surface. Although some previous reports did not mention the number of cRGD ligands on their carriers surfaces, the nanocarriers reported by Schiffelers *et al* [19], Mulder *et al* [21], and Cai *et al* [25], could fulfill the requirements of the above equation. FCS measurements revealed that the numbers of cRGD on cRGD-PICsomes were 6400 for the 100%-cRGD PICsomes, 2600 for the 40%-cRGD PICsomes, and 1500 for the 20%-cRGD PICsomes (table S1). In addition, we calculated the distance between two cRGD molecules on the surface of a single PICsome, and the average distances were estimated to be 2.2 nm for 100%-cRGD PICsomes, 3.5 nm for 40%-cRGD PICsomes, and 4.6 nm for 20%-cRGD PICsomes (table S1). These results indicate that the numbers of cRGDs on the surface of each PICsome was sufficient and the cRGD−cRGD distances were smaller than the size of the integrin head for multivalent binding to occur between single cRGD-linked PICsome and multiple integrin molecules. Taking into account these multivalent bindings, it can be presumed that tuning the cRGD density and carrier size enable to improve the accumulation in targeted sites such as the neovasculature. However, it is known that the multivalent bindings lead to lowered dissociation constants [37], and this might affect the restriction of extravasation and/or the translocation of the nanocarriers. Moreover, as we described in section 3.3, controlling cRGD-density enables to reduce the accumulation of nanocarriers in normal tissues, and enhance tumor accumulation. Thus, designing and tuning of cRGD-linked nanocarriers with appropriate cRGD-density and size is fundamentally important. Although further detailed studies are required to explain the whole phenomenon, our findings indicate that cRGD-linked PICsomes with 40% cRGD-density are likely a contributing factor to their localization in the endothelial layer without penetration into the tumor parenchyma and that these characteristics can be advantageous in drug delivery applications such as neovascular imaging.

### 3.5. Preparation of contrast agent-loaded cRGD-PICsomes

**Figure 8.**
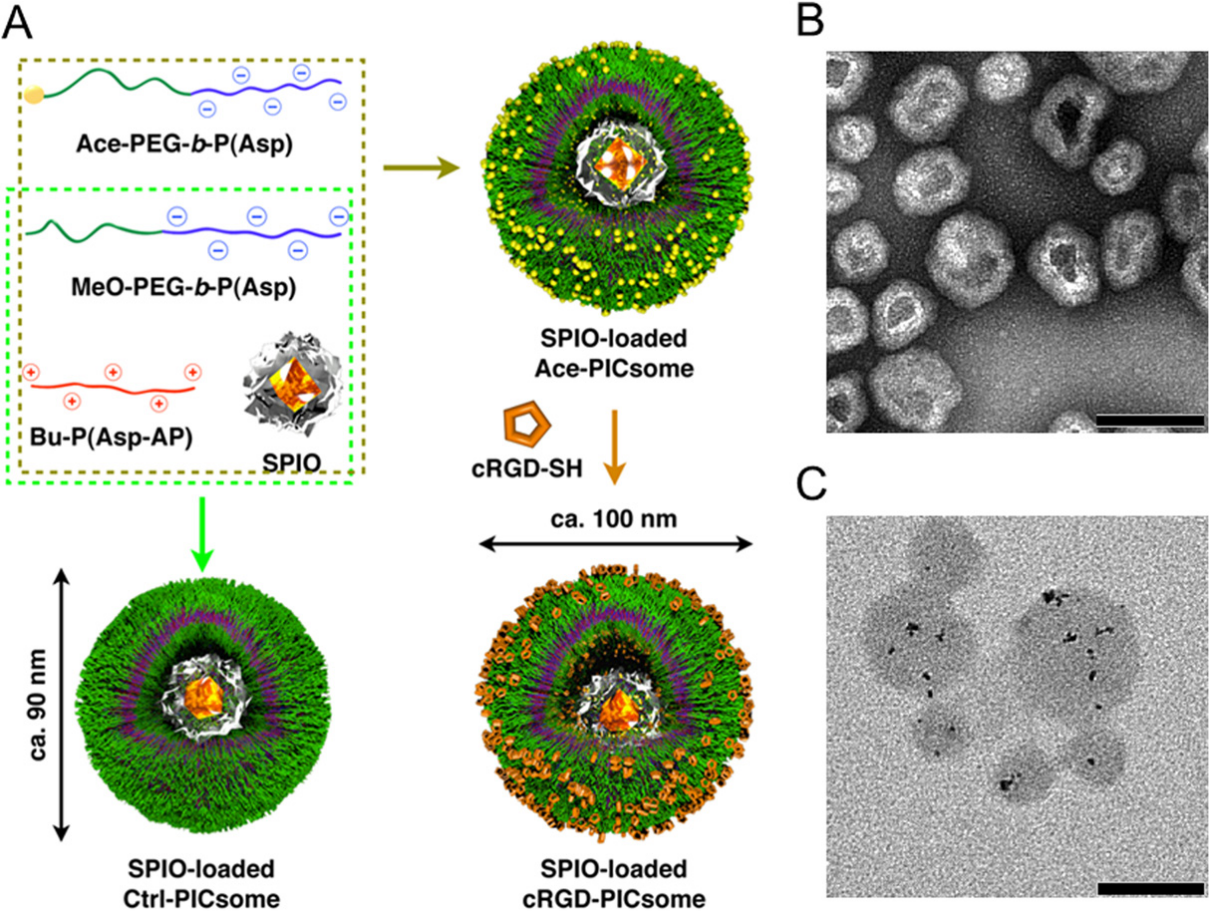
Schematic representation of the method used to prepare SPIO-loaded PICsomes and TEM images of the resulting PICsomes. (A) Schematic representation of the protocol used to prepare SPIO-loaded PICsomes. (B) TEM image of SPIO-loaded 40%-cRGD-PICsomes stained with uranyl acetate. (C) TEM image of unstained SPIO-loaded PICsomes. Scale bars = 100 nm in all images.

PICsomes can incorporate substances into their inner hollow spaces for delivery to angiogenic sites navigated by the cRGD ligand on the PICsome surface. To use these characteristics for medical applications such as tumor diagnosis, we prepared cRGD-PICsomes that incorporated SPIO nanoparticles (ferucarbotran) approved by the US Food and Drug Administration [7] (figure 8(A)). SPIO is a widely used reagent for negative contrast MRI; therefore, SPIO-loaded cRGD-PICsomes could provide clear MR images and related information for tumor diagnosis because of their characteristic features such as their ability to survive for long periods in the circulation and their targeting ability.

The structure of the SPIO-loaded cRGD- and Ctrl-PICsomes was confirmed using TEM. As shown in figure 8(B), SPIO incorporation did not affect the morphology of PICsomes stained with uranyl acetate. We then performed TEM without uranyl acetate staining, and incorporated SPIO were observed in the inner hollow spaces of the PICsomes (black spots in figure 8(C)). Moreover, EDS revealed the presence of Fe signals (figures S7 and S8), indicating the successful incorporation of SPIO into PICsomes. Other characteristics, such as size, zeta-potential, and Fe concentration, are listed in table S2. We also measured *R*_2_, which is the reciprocal of the transverse relaxation time (*T*_2_) of water protons (^1^H), using *in vitro* MRI. Similar concentration-dependent increases in *R*_2_ values and *R*_2_ color mapping were observed for SPIO-loaded cRGD-PICsomes, Ctrl-PICsomes and ferucarbotran (Resovist), suggesting that they have comparable potential as contrast agents (figure S9).

### 3.6. *In vivo* MRI of orthotopic glioblastoma

**Figure 9.**
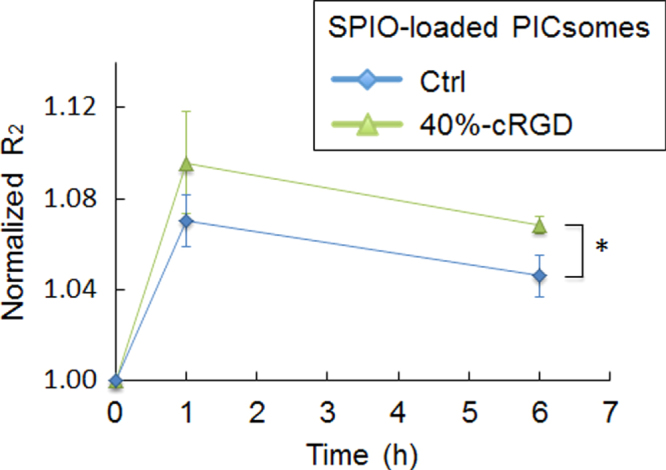
Changes in the normalized *R*_2_ value over time in orthotopic tumors in the brain. Quantitative *R*_2_ values were normalized to the value obtained before PICsome administration. The data were analyzed using Student’s *t*-test. Data are presented as the mean ± s.e.m. (*n* = 4 for 40%-cRGD PICsomes, and *n* = 3 for Ctrl-PICsomes). ∗*P* < 0.05.

**Figure 10.**
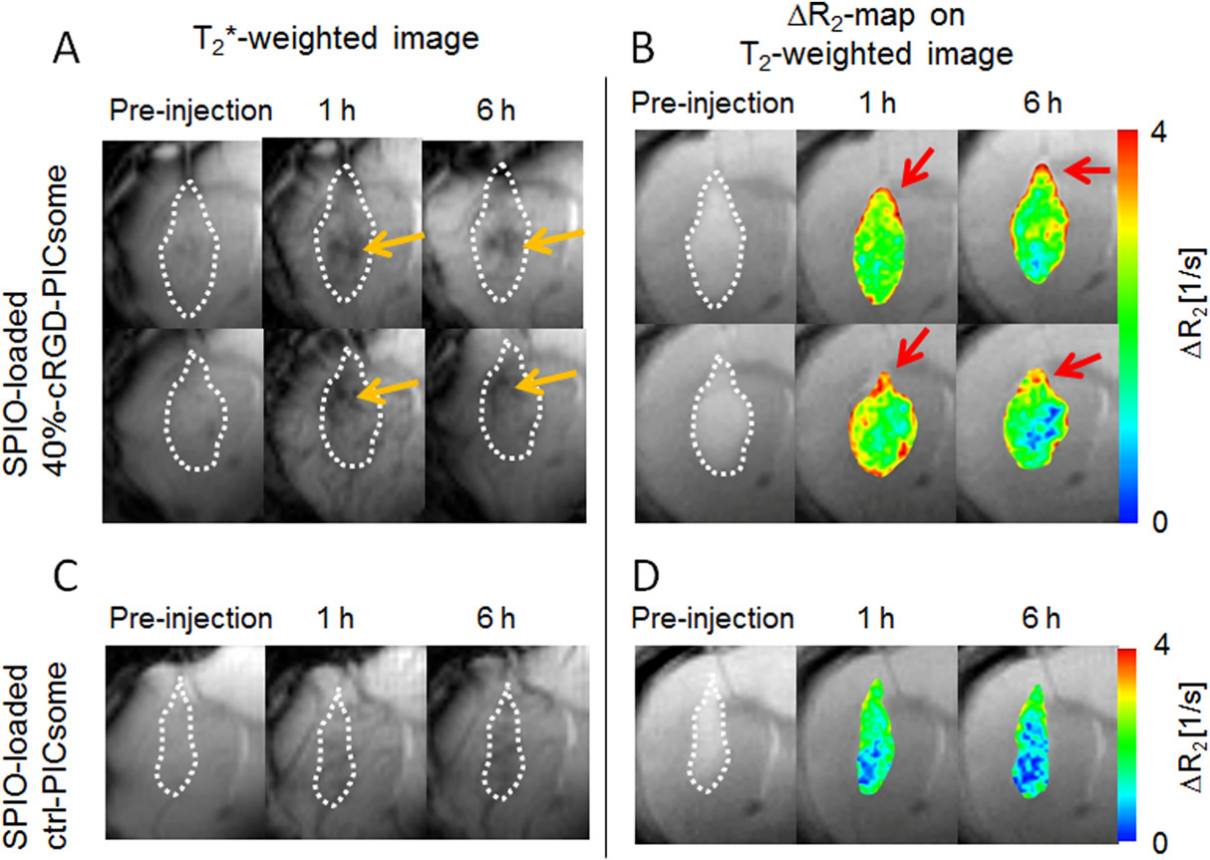
MRI of U87MG orthotopic glioblastoma with SPIO-loaded PICsomes. *T*_2_∗-weighted MR images of (A) 40%-cRGD-PICsomes (*n* = 2) and (C) Ctrl-PICsomes (*n* = 2) before, and 1 and 6 h after administration. (B) *ΔR*_2_ maps in the tumor 1 and 6 h after PICsome administration compared with pre-administration. Tumor lesions were identified using *T*_2_-weighted MRI, and are circled with white dashed lines. Orange arrows represent a reduction of the signal of the tumors. Red arrows represent the sites with significantly increased *ΔR*_2_ values.

Orthotopic U87MG glioblastoma was selected as a model tumor for MR imaging by SPIO-loaded cRGD-PICsomes. Both SPIO-loaded 40%-cRGD-PICsomes and Ctrl-PICsomes were administered at a dose of 40 *μ*mol Fe kg^−1^ for MRI measurements. Changes in the normalized *R*_2_ values in tumor lesions over time are shown figure 9. A significant increase in normalized *R*_2_ values was observed in SPIO-loaded cRGD-PICsomes compared with Ctrl-PICsomes. Furthermore, the unique features of SPIO-loaded cRGD-PICsomes are demonstrated in the *T*_2_∗-weighted GE images shown in figure 10(A). Specifically, glioblastoma lesions treated with cRGD-PICsomes underwent a strong reduction in signal at 1 h, which was maintained even after 6 h (figure 10(A), orange arrow). Interestingly, the *ΔR*_2_ maps clearly show intensified signal in the tumors of mice that were administered cRGD-PICsomes (figure 10(B)). In particular, there was a significant increase in the *ΔR*_2_ value at the rim of the glioblastoma (figure 10(B), red arrows). Because angiogenesis is correlated with tumor malignancy and is active at the tumor periphery [38], these contrast images suggest the selective binding of SPIO-loaded cRGD-PICsomes to the overexpressed *α*_V_*β*_3_ and *α*_v_*β*_5_ integrins in the tumor neovasculature [16, 23, 24, 38], which is consistent with the IVCLSM images shown in figure 6. In contrast, MR images obtained from SPIO-loaded Ctrl-PICsomes were less clear (figure 10(C)), and the *ΔR*_2_ value was also lower than that of cRGD-PICsomes (figure 10(D)). These data highlight the potential application of PICsomes in the medical field and suggest that cRGD installation might be beneficial for targeting the neovasculature, allowing enhanced MR images even in an orthotopic glioblastoma model.

## 4. Conclusions

In the present study, we developed a sound scheme for quantitatively attaching cRGD ligands onto the surface of PICsomes, and demonstrated the feasibility of targeting tumors through the selective binding of cRGD-linked PICsomes to *α*_V_*β*_3_ and *α*_v_*β*_5_ integrins overexpressed in the tumor neovasculature. Then, we assessed the efficacy of SPIO-loaded cRGD-PICsomes for MRI in an orthotopic glioblastoma model, demonstrating their utility to image sites of angiogenesis. Because vasculature formation is correlated intimately with tumor malignancy, tumor imaging using neovascularly targeted PICsomes should be a promising tool for accurate diagnosis of malignant tumors. This study highlights the importance of the tunable design of PICsomes with integrated functionalities, including stealth property to exert longevity in the blood circulation, while high targetability to the particular site in tumor, in this case, a site undergoing vigorous angiogenesis. Accordingly, these functionalized PICsomes have great potential for the use in drug delivery and imaging, even against solid tumors that have tight vascular/tumor barrier such as brain tumors.
